# Differences in treatment of stage I colorectal cancers: a population-based study of colorectal cancers detected within and outside of a screening program

**DOI:** 10.1055/a-2173-5989

**Published:** 2023-11-07

**Authors:** Esther Toes-Zoutendijk, Emilie C. H. Breekveldt, Lisa van der Schee, Iris D. Nagtegaal, Marloes A. G. Elferink, Iris Lansdorp-Vogelaar, Leon M. G. Moons, Monique E. van Leerdam

**Affiliations:** 1 Department of Public Health, Erasmus University Medical Center, Rotterdam, The Netherlands; 2 Department of Gastrointestinal Oncology, Netherlands Cancer Institute – Antoni van Leeuwenhoek Hospital, Amsterdam, The Netherlands; 3 Department of Gastroenterology and Hepatology, University Medical Center Utrecht, Utrecht, The Netherlands; 4 Department of Pathology, Radboud University Medical Center, Nijmegen, The Netherlands; 5 Department of Research and Development, Netherlands Comprehensive Cancer Organization, Utrecht, The Netherlands; 6 Department of Gastroenterology and Hepatology, Leiden University Medical Center, Leiden, The Netherlands

## Abstract

**Background** Screen-detected colorectal cancers (CRCs) are often treated less invasively than stage-matched non-screen-detected CRCs, but the reasons for this are not fully understood. This study evaluated the treatment of stage I CRCs detected within and outside of the screening program in the Netherlands.

**Methods** Data from the Netherlands Cancer Registry for all stage I CRCs diagnosed between January 1, 2008 and December 31, 2020 were analyzed, comparing patient, tumor, and treatment characteristics of screen-detected and non-screen-detected stage I CRCs. Multivariable logistic regression was used to assess the association between treatment (local excision only vs. surgical oncologic resection) and patient and tumor characteristics, stratified for T stage and tumor location.

**Results** Screen-detected stage I CRCs were relatively more often T1 than T2 compared with non-screen-detected stage I CRCs (66.9 % vs. 53.3 %; *P* < 0.001). When only T1 tumors were considered, both screen-detected colon and rectal cancers were more often treated with local excision only than non-screen-detected T1 cancers (odds ratio [OR] 2.19, 95 %CI 1.93–2.49; and OR 1.29, 95 %CI 1.05–1.59, respectively), adjusted for sex, tumor location, lymphovascular invasion (LVI) status, and tumor differentiation.

**Conclusions** Less invasive treatment of screen-detected stage I CRC is partly explained by the higher rate of T1 cancers compared with non-screen-detected stage I CRCs. T1 stage I screen-detected CRCs were also more likely to undergo less invasive treatment than non-screen-detected CRCs, adjusted for risk factors such as LVI and tumor differentiation. Future research should investigate whether the choice of local excision was related to unidentified cancer-related factors or the expertise of the endoscopists.

## Graphical abstract

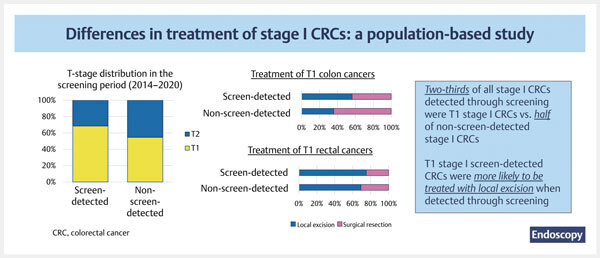

## Introduction

In the last decades, many countries have implemented colorectal cancer (CRC) screening programs to reduce the incidence and mortality of CRC 1 2 . Reductions in CRC incidence and mortality can be achieved by the removal of precursor lesions and detection of CRC at an early stage. Early-stage CRCs have better survival rates and require less invasive treatment than advanced-stage CRCs. Therefore, a stage shift resulting from the implementation of CRC screening implies the need for less invasive treatment of CRC and a decrease in mortality may be expected 3 4 5 6 .

In previous studies it has been shown that screen-detected CRCs are more likely to be treated less invasively (i. e. by local excision only) than those detected outside of a CRC screening program (non-screen-detected CRCs) 6 7 . Remarkably, this phenomenon also occurred when treatment for only stage I CRCs was considered, with significantly more local excisions when these CRCs were detected through screening 6 . The reasons why early-stage screen-detected CRCs are treated by less invasive methods compared with non-screen-detected CRCs, even if they are diagnosed at the same stage, are still not fully understood.

Several hypotheses could account for the observed difference in treatment within stage I CRCs. First, there may be an uneven T1/T2 distribution for stage I CRCs detected within and outside of the CRC screening program. If proportionately more T1 stage I CRCs are detected by screening, this may lead to a higher rate of local excision only for screen-detected rather than non-screen-detected stage I CRCs 8 . Second, the location of screen-detected CRCs differs from non-screen-detected CRCs; screening detects relatively more left-sided colon cancers 6 9 10 . As left-sided colon cancers are more easily removed than right-sided colon cancers, we hypothesize that the higher proportion of local excisions for screen-detected stage I CRCs is due to the unequal distribution of cancers in the colon and rectum 11 . Third, the presence of prognostic factors (i. e. resection margin status, lymphovascular invasion (LVI), grade of differentiation, or tumor budding) may drive the decision to refer for (additional) surgical oncologic resection 12 13 14 15 . If these prognostic factors differ between screen-detected and non-screen-detected stage I CRCs, this is likely to result in different rates of surgical oncologic resection. Finally, other (nontumor-related) factors may have determined the decision to refer for surgical oncologic resection.

The aim of this study was to describe the treatment of stage I CRCs detected within and outside of the CRC screening program in the Netherlands on a population level. Furthermore, we aimed to determine to what extent patient and tumor characteristics explain the difference in treatment of patients with stage I CRC.

## Methods

### Dutch CRC screening program

Since 2014, the nationwide CRC screening program has been gradually implemented in the Netherlands 16 . The target population of the program is men and women aged 55–75. The target population is invited to undergo screening biennially and receives an invitation letter including a fecal immunochemical test (FIT; FOB-Gold; Sentinel Diagnostics, Milan, Italy). Individuals with a positive FIT receive an invitation to undergo colonoscopy. Individuals with a negative FIT are invited for repeat FIT screening after 2 years.

### Databases

All patients diagnosed with stage I CRCs between January 1, 2008 and December 31, 2020 were selected from the Netherlands Cancer Registry (NCR); the NCR registers all newly diagnosed malignancies in the Netherlands. Data from the NCR include: patient characteristics (sex and age) and tumor characteristics (incidence year; tumor, node, metastasis (TNM)-staging; location; histology; LVI; tumor differentiation; and treatment). Data are linked to the Dutch nationwide pathology databank (PALGA) to identify whether these CRCs were screen-detected or non-screen-detected tumors (99.2 % of patients from the NCR could be reliably matched). When patients had multiple primary CRCs, the tumor with the first incidence date was included in the analyses. Patients with synchronous CRCs (i. e. more than one tumor with the same date of diagnosis) were excluded from the analyses, as their treatment differs from patients with one tumor.

### Definitions

The prescreening era was defined as the incidence years 2008–2013. The screening era was defined as the incidence years 2014–2020. Only individuals aged ≥ 55 and < 80 years were included to ensure a similar age distribution of individuals with screen-detected and non-screen-detected CRCs. The upper age limit of 80 years was chosen to allow for a delay in screening invitation, return of the FIT, and/or CRC diagnosis.

CRC stage was classified using the TNM staging system effective at the time of diagnosis (6th, 7th, or 8th editions) 17 18 19 . Patients who received neoadjuvant treatment were excluded (1956 [8.0 %] stage I CRCs) as such treatment may interfere with the accurate evaluation of the initial staging. Stage I CRCs were defined as T1Nx/N0 and T2Nx/N0 tumors. Hereafter, we will refer to T1Nx/N0 CRCs as T1 CRC and to T2Nx/N0 CRCs as T2 CRC. Location was defined as follows: right-sided colon (cecum to transverse colon, C18.0, C18.2–C18.4), left-sided colon (splenic flexure to rectosigmoid, C18.5–C18.7, C19), rectum (C20), and overlapping and unspecified (C18.8–C18.9). Appendiceal cancers (C18.1) were excluded from this study. LVI was defined as (suspicion of) invasion of the cancer cells into either the blood or lymphatic vessels. A three-tiered classification system was applied for grade: well (grade 1), moderately (grade 2), and poorly differentiated (grade 3).

Local excision included endoscopic resection, transanal endoscopic microsurgery (TEM), or transanal minimally invasive surgery (TAMIS). Surgical oncologic resection included all other forms of resection. When local excision was followed by surgical oncologic resection (secondary surgical oncologic resection), this was considered surgical oncologic resection.

### Outcomes

Primary outcomes included the incidence and treatment of screen-detected versus non-screen-detected stage I CRCs, as a whole and separately for T1/T2 tumors. Secondary outcomes included tumor characteristics and factors associated with the treatment of screen-detected vs. non-screen-detected stage I CRCs.

### Statistical analyses

Chi-squared testing was used to compare the characteristics of screen-detected and non-screen-detected stage I CRCs. The Wilcoxon–Mann–Whitney *U* test was used to compare the median ages of patients with screen-detected and non-screen-detected cancers. Two-sided *P* values < 0.05 were considered statistically significant.

Join-point regression analyses were performed to evaluate changes in treatment by calculation and comparison of the annual percentage change (APC) in treatment of T1 CRC. Two join points were used as the maximum number of join points with a minimum difference of 0.5 percentage points. Multivariable logistic regression analyses were used to assess the association between treatment (local excision only versus surgical oncologic resection) and mode of detection (screen-detected vs. non-screen-detected), sex, age category, LVI status, tumor differentiation, and location of the tumor. The presence of multicollinearity was checked using the variance inflation factor (VIF). VIF values ≥ 5 were considered to indicate collinearity and highly correlated variables were removed from the model. Separate models were constructed for T1 colon and T1 rectal cancers. As almost all T2 CRCs were treated by surgical oncologic resection, the number of patients with T2 CRCs treated by local excision only was insufficient to perform join-point and logistic regression analyses.

Join point regression analyses were performed using Join point regression software of the US National Cancer Institute. All other analyses were performed using R version 4.0.2.

### Sensitivity analysis

A sensitivity analysis was performed to rule out selection bias in the referral of screen-detected versus non-screen-detected stage I (T1) CRCs. Selection bias may be present if a higher proportion of T1 cancers in one group is less often treated by surgical oncologic resection and is therefore not examined for lymph node metastases (LNM). We examined data from all T1 tumors diagnosed from 2014 to 2020 (stage I and IIIa/b) ( **Table 1 s** , see online-only Supplementary material). We compared the treatment of screen-detected T1 tumors with the treatment of non-screen-detected T1 tumors. Where there are similar treatments for screen-detected stage I T1 CRCs and non-screen-detected T1 CRCs, biases in the selection and conclusions with regard to the treatment of stage I T1 CRCs are less likely to arise.

## Results

In the period 2008–2020, 22 433 stage I CRCs were identified in patients aged 55–79 years. Of these cancers, 6130 (27.3 %) were detected in the period prior to the implementation of screening (2008–2013). In the screening period (2014–2020), 6188 (27.6 %) screen-detected and 10 115 (45.1 %) non-screen-detected stage I CRCs were identified. A total of 277 (1.2 %) CRCs with unknown T stage were excluded from the analyses.

### Patient and tumor characteristics for stage I CRCs

**Fig. 1:**
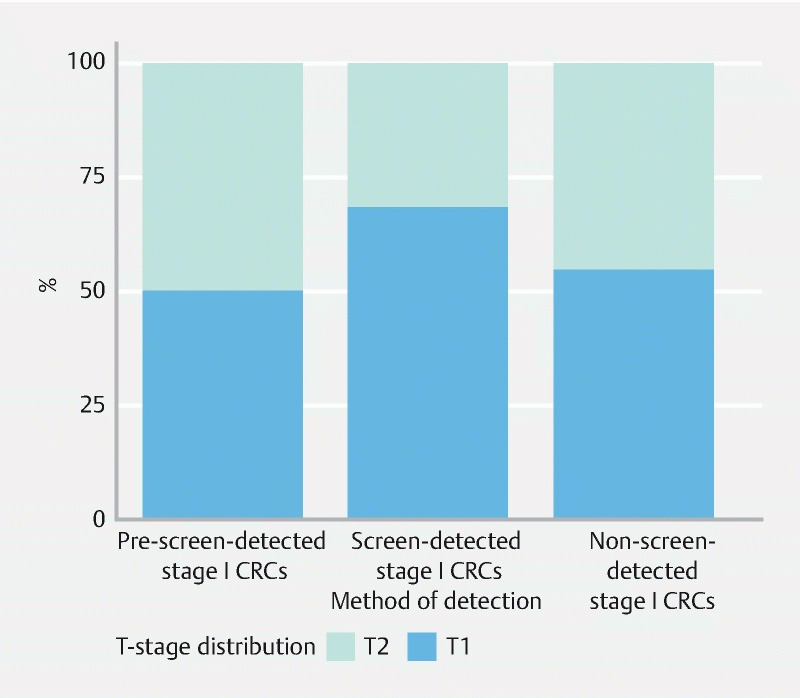
T-stage distribution of stage I colorectal cancers (CRCs) by method of detection. Pre-screening CRCs were not taken into account in statistical analysis.

In the prescreening era, stage I CRCs were comprised of 50.4 % (n = 3052) T1 CRCs and 49.6 % (n = 3008) T2 CRCs ( Fig. 1 ). In the screening era, screen-detected stage I CRCs comprised 68.5 % (n = 4172) T1 CRCs and 31.5 % (1922) T2 CRCs. Non-screen-detected stage I CRCs consisted of 54.6 % (n = 5464) T1 CRCs and 45.4 % (4538) T2 CRCs. The T1/T2 proportion differed significantly between screen-detected and non-screen-detected stage I CRCs ( *P* < 0.001). Patients with screen-detected CRCs were slightly younger than patients with non-screen-detected CRCs ( *P* < 0.001) ( Table 1 ). For all stage I CRCs in the screening era, regardless of the mode of detection, the majority of patients were male, with the largest proportion of men in the T1 stage I CRC group ( *P* < 0.001) ( Table 1 ).

**Table 1:** Characteristics of T1 /T2 stage I colorectal cancers (CRCs) detected within and outside of the CRC screening program.

	T1 CRC		*P* value	T2 CRC		*P* value
	Screen-detected (n = 4172)	Non-screen-detected (n = 5464)		Screen-detected (n = 1922)	Non-screen-detected (n = 4538)	
Age, median (IQR), years	67 (63–73)	69 (63–74)	< 0.001	67 (63–73)	70 (64–74)	< 0.001
Sex, n (%)			< 0.001			0.76
Men	2643 (63.4)	3230 (59.1)		1108 (57.6)	2596 (57.2)	
Women	1529 (36.6)	2234 (40.9)		814 (42.4)	1942 (42.8)	
Location 1			< 0.001			< 0.001
Left-sided	2585 (62.9)	2291 (42.7)		749 (39.3)	1412 (31.5)	
Right-sided	528 (12.8)	1428 (26.6)		695 (36.4)	1921 (42.9)	
Rectum	999 (24.3)	1643 (30.6)		463 (24.3)	1149 (25.6)	
LVI 1			0.33			0.75
No	2824 (67.7)	3511 (64.3)		1409 (73.3)	2969 (65.4)	
Yes	473 (11.3)	550 (10.1)		180 (9.4)	393 (8.7)	
Unknown	875 (21.0)	1403 (25.7)		333 (17.3)	1176 (25.9)	
Differentiation 2			0.23			0.57
Grade 1	40 (1.0)	286 (5.2)		21 (1.1)	128 (2.8)	
Grade 2	3749 (89.9)	4528 (82.9)		1739 (90.5)	3999 (88.1)	
Grade 3	79 (1.9)	121 (2.2)		69 (3.6)	147 (3.2)	
Unknown/NA	304 (7.3)	529 (9.7)		93 (4.8)	264 (5.9)	

IQR, interquartile range; LVI, lymphovascular invasion; NA, not applicable.

1 Category “unknown” was not taken into account for chi-squared testing.

2 Chi-squared testing for grade 1 + grade 2 vs. grade 3 (category “unknown” was not taken into account).

In the prescreening era, a total of 33.6 % (n = 2059) stage I cancers were right-sided, 47.1 % (n = 2886) were left-sided, and 17.2 % (n = 1051) were rectal cancers. In the screening era, location significantly differed between screen-detected and non-screen-detected stage I CRCs; screen-detected stage I cancers were more often located in the left side of the colon (54.7 %, n = 3386) than non-screen-detected stage I cancers (37.0 %, n = 3747; *P* < 0.001).

No differences in LVI status were observed for screen-detected and non-screen-detected CRCs. For T1 CRCs, LVI was present in 67.7 % (n = 2824) of screen-detected CRCs compared with 64.3 % (n = 3511) of non-screen-detected CRCs ( *P* = 0.33) ( Table 1 ). For T2 CRCs, LVI was present in 73.3 % (n = 1409) of screen-detected CRCs vs. 65.4 % (n = 2969) of non-screen-detected CRCs ( *P* = 0.75). The majority of both screen-detected and non-screen-detected stage I CRCs showed moderate differentiation in both T1 ( *P* = 0.23) and T2 ( *P* = 0.57) CRCs ( Table 1 ).

### Treatment of stage I colon cancers

**Fig. 2:**
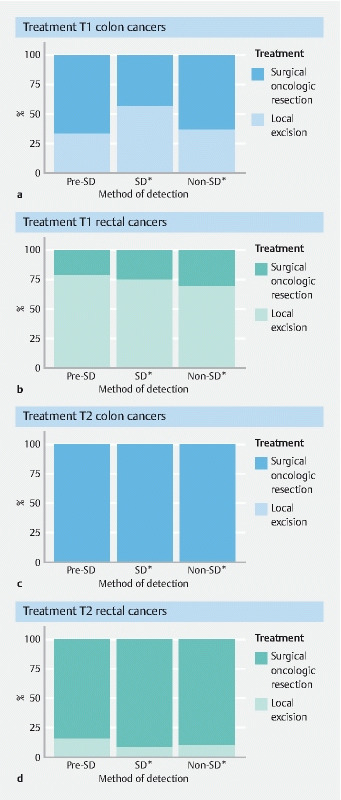
Treatment of stage I colon and rectal cancers by T stage and method of detection. SD: screen-detected *statistically significant difference

**Fig. 3:**
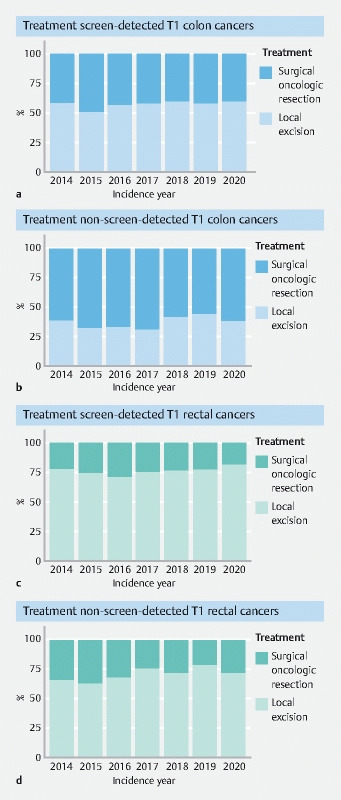
Treatment of T1 colon and rectal cancers from 2014–2020 by method of detection.

In the prescreening era, 33.1 % (n = 746) of T1 and 0.4 % (n = 11) of T2 colon cancers were treated by local excision only. In the screening era, local excision was performed on 56.4 % (n = 1753) of screen-detected vs. 35.9 % (n = 1332) of non-screen-detected T1 colon cancers ( *P* < 0.001) ( Fig. 2a ). This difference was not observed in T2 colon cancers; the majority of patients were treated by surgical oncologic resection (99.6 %) and no significant differences were observed in treatment between screen-detected and non-screen-detected T2 colon cancers ( *P* = 0.89) ( Fig. 2b ). The proportion of T1 colon cancers treated by local excision slightly increased over time in screen-detected colon cancers (APC 1.5 %, 95 %CI 1.4 % to 4.4 %) ( Fig. 3a ), as well as in non-screen-detected colon cancers (APC 3.2 %, 95 %CI 3.1 % to 9.9 %) ( Fig. 3b ). However, no significant changes were observed in trends and no join points were identified.

### Treatment of stage I rectal cancers

In the prescreening era, 79.1 % (n = 564) of T1 and 15.2 % (n = 47) of T2 rectal cancers were treated by local excision only. In the screening era, local excision was performed in 75.2 % (n = 751) of screen-detected vs. 69.2 % (n = 1135) of non-screen-detected T1 rectal cancers ( *P* < 0.001) ( Fig. 2c ). Again, treatment of T2 rectal cancers did not significantly differ: 91.8 % (n = 424) of screen-detected and 90.0 % (n = 1033) of non-screen-detected T2 rectal cancers were treated by surgical oncologic resection ( *P* = 0.51) ( Fig. 2 d ). In the screening era, the proportion of T1 screen-detected rectal cancers treated by local excision decreased until 2016 and significantly increased after this: APC 2014–2016, −3.9 % (95 %CI −12.4 % to 5.4 %); APC 2016–2020, 3.2 % (95 %CI 0.7 % to 5.8 %) ( Fig. 3c ). The proportion of T1 non-screen-detected rectal cancers treated by local excision increased from 2014 onwards; however, this trend was nonsignificant and no join points were identified (APC 2.7 %, 95 %CI −0.6 % to 6.2 %) ( Fig. 3 d ).

### Factors associated with the treatment of T1 tumors

In T1 rectal cancers, women had a higher likelihood of undergoing surgical oncologic resection than men (odds ratio [OR] 1.26, 95 %CI 1.03 to 1.55) ( Table 2 ). Patients with LVI were more likely to undergo surgical oncologic resection in both T1 colon (OR 3.15, 95 %CI 2.61 to 3.81) and T1 rectal cancers (OR 1.55, 95 %CI 1.17 to 2.03). Among patients diagnosed with T1 colon cancers, those with right-sided tumors were significantly more likely to undergo surgical oncologic resection than those with left-sided tumors (OR 4.20, 95 %CI 3.61 to 4.90). Patients with poorly differentiated tumors were also more often treated by surgical oncologic resection compared with patients with well-differentiated tumors, in both T1 colon cancers (OR 6.96, 95 %CI 3.63 to 12.85) and T1 rectal cancers (OR 3.19, 95 %CI 1.26 to 8.43).

**Table 2:** Multivariable logistic regression analyses for the association between treatment and patient and tumor characteristics for the separate T1 colon and T1 rectal cancer models.

	Odds ratio (95 %CI)	
	T1 colon cancers	T1 rectal cancers
Sex		
Male	1	1
Female	1.03 (0.91–1.18)	1.26 (1.03–1.55)
Age group, years		
55–59	1	1
60–64	1.21 (0.98–1.51)	1.01 (0.72–1.43)
65–69	1.12 (0.91–1.37)	1.15 (0.83–1.59)
70–74	0.92 (0.74–1.13)	0.84 (0.60–1.17)
75–79	0.75 (0.60–0.94)	1.10 (0.76–1.59)
Lymphovascular invasion		
No	1	1
Yes	3.15 (2.61–3.81)	1.55 (1.17–2.03)
Location		
Left	1	N/A
Right	4.20 (3.61–4.90)	N/A
Tumor differentiation		
Grade 1	1	1
Grade 2	1.58 (1.06–2.35)	1.32 (0.69–2.68)
Grade 3	6.96 (3.63–12.85)	3.19 (1.26–8.43)
Detection		
Screen-detected	1	1
Non-screen-detected	2.19 (1.93–2.49)	1.29 (1.05–1.59)

Upon adjusting for the previously mentioned risk factors, non-screen-detected T1 colon cancers had twice the likelihood of undergoing surgical oncologic resection in comparison with screen-detected T1 colon cancers (OR 2.19, 95 %CI 1.93 to 2.49). A similar association was observed for T1 rectal cancers; however, the magnitude of the effect was smaller (OR 1.29, 95 %CI 1.05 to 1.59).

### Sensitivity analysis

When considering all T1 CRCs (stage I and stage III; n = 10 245), 6.3 % (n = 278) of screen-detected T1 CRCs were stage III vs. 6.1 % (n = 355) of all non-screen-detected T1 CRCs ( *P* = 0.81) ( **Table 1 s** ). Local excision only was performed in 57.2 % (n = 2543) of screen-detected T1 CRCs versus 43.6 % (n = 2627) of non-screen-detected T1 CRCs ( *P* < 0.001).

## Discussion

The aim of this study was to describe the treatment of stage I CRCs detected within and outside of the CRC screening program in the Netherlands on a population level. Furthermore, we aimed to determine to what extent patient and tumor characteristics explain the difference in the treatment of patients with stage I CRC. We showed that two-thirds of all stage I CRCs detected through screening were T1 stage I CRCs. In contrast, only half of non-screen-detected stage I CRCs were T1 stage I. In addition, when only the T1 stage I colon and rectal cancers were considered, these were more likely to be treated with local excision when detected through screening.

We hypothesized that the less invasive treatment of screen-detected compared with non-screen-detected stage I CRCs could be explained by the unequal T1/T2 distribution within stage I CRCs. Screen-detected CRCs had a relatively higher proportion (13.6 percentage points) of T1 cancers compared with non-screen-detected CRCs. These findings suggest that the unequal T1/T2 distribution within stage I CRCs is an important explanation for the more frequent use of less invasive treatment for screen-detected stage I CRCs, as T1 tumors lacking high risk features for LNM can be safely treated by local excision. Fewer surgical oncologic resections may however have caused an underestimation of the T1 stage III CRCs owing to there being fewer lymph node dissections. However, as shown in the sensitivity analysis, the distribution between T1N0 and T1N + for screen-detected and non-screen-detected CRCs was comparable, with only a 0.13 percentage point difference in T1N + CRCs detected by screening. Therefore, the lower number of surgical oncologic resections among screen-detected T1 CRCs cannot be explained by the distribution of T1N0 and T1N + tumors.

Several studies have compared rates of local excision and surgical oncologic resection of T1 CRCs. In the sensitivity analysis of all T1 CRCs in the current study, local excision rates were higher for screen-detected T1 CRCs (55.5 %) than for non-screen-detected T1 CRCs (41.5 %). These observed rates were higher than those found in four other studies from Italy (23.1 %), the UK (31 %), the USA (35.5 %), and France (21.3 %) 11 20 21 22 . The reason for this is not fully understood, but it may be due to improvements in endoscopic techniques in recent years, making it easier to remove T1 CRCs through local excision only 23 . Notably, some of the studies mentioned were conducted many years ago, so may not reflect current trends in the management of T1 CRCs.

In addition to the explanation of less invasive treatment by the more favorable distribution of T1 and T2 stages for screen-detected stage I CRCs, we also observed differences in the treatment for screen-detected and non-screen-detected T1 stage I CRCs for both colon and rectal cancers. Non-screen-detected T1 colon cancers were twice as likely to be treated with surgical oncologic resection as were screen-detected T1 colon cancers, even after adjustment for well-known confounders (e. g. LVI and tumor differentiation). The same was true for rectal cancers, but to a lesser extent.

Explanations for this phenomenon are unknown, but it may be related to the level of experience of endoscopists in assessing and/or removing malignant polyps in the right- and left-sided colon. Endoscopists first need to fulfill the eligibility quality criteria to be able to perform colonoscopies within the Dutch CRC screening program. Additionally, there are annual audits and colonoscopy results are benchmarked within the national screening program to ensure high quality endoscopies (i. e. adenoma detection rate of ≥ 40 %, cecum intubation rate of ≥ 95 %) 24 . This may bias the screening program towards having more endoscopists who can assess polyps for local excision. Unfortunately, we were not able to distinguish whether endoscopies were performed by an expert endoscopist, or in an expert center or a general endoscopy center, which may also be related to the performance of the endoscopist, as well as data on resection margin or en bloc resection. In addition to endoscopist experience, observed treatment differences may be related to other tumor characteristics (i. e. morphology, residual tumor status, size of the tumor, and tumor budding) of CRCs that were not reported or other patient-related characteristics. For example, in our study we observed that men with rectal cancers were more often treated with local excision only compared with women.

Another explanation for more local excisions in the screen-detected stage I CRC group is tumor location. Among all T1 colon cancers, right-sided tumors were more often treated by surgical oncologic resection. Relatively more left-sided colon cancers are detected through FIT-based screening than outside of the screening program 6 . This partly explains the larger proportion of local excisions only in patients with screen-detected T1 colon cancers, as left-sided and rectal tumors can more often be removed with noninvasive treatment methods. Other characteristics of the patient or tumor may have also driven the treatment decision. LVI status and poor differentiation grade were associated with higher rates of surgical oncologic resection, which is in line with our expectations, the literature, and Dutch guidelines because of the risk of LNM 15 25 26 . However, given the similar distribution of LVI and differentiation grade in both screen-detected and non-screen-detected T1 CRCs, this cannot explain the difference in treatment.

Despite the significant association between LVI and surgical oncologic resection, the proportion of tumors with LVI (i. e. 11 % of T1 colon cancers) was much lower than the expected 18 %–30 % found in the literature 27 28 . An explanation for this could be the significant number of patients (approximately 25 %) with unknown LVI status, which has also been observed in other population-based studies using national databases. We do not however anticipate a difference in the LVI status between the unknown cases in the screen-detected and non-screen-detected groups.

A major strength of this study is its large sample size, including all stage I CRCs diagnosed between 2008 and 2020, using nationwide population-based cancer registry data. The large sample size enabled us to carry out multiple subgroup analyses. By using a nationwide database, we could include all CRCs regardless of which hospital the diagnosis was made in (i. e. academic medical centers, teaching hospitals, or peripheral/general hospital).

The main limitation of this study is the absence of data on relevant risk factors (i. e. morphology, residual tumor status, and tumor budding) that could have driven the choice of treatment. This is due to the fact that some of these factors were only partly available in the NCR, while others were not registered until a later phase of the study. Moreover, complete information on co-morbidities or patient preferences is only accessible for a proportion of the patients included in the national database. Because these risk factors are not assessed and/or recorded in a standardized manner or available on a population level, we did not incorporate them in the statistical analyses. Standardized assessment and reporting of relevant risk factors is recommended.

Furthermore, we encountered the difficulty of distinguishing between secondary oncologic resections and direct referral for oncological resection, as the linkage between local excisions followed by surgical oncologic resection was not consistently reliable. Nonetheless, since 2019, this link has become more dependable, potentially enabling a subgroup analysis to be carried out in the future.

The difference in treatment between screen-detected and non-screen-detected stage I CRCs cannot be fully explained by the available risk factors in this study, suggesting that the mode of detection partially drives the more favorable treatment. The greater competence of endoscopists in identifying and assessing potentially malignant polyps to be eligible for local excision, along with the better health of the screened population may contribute to this difference. Many endoscopy centers performing local excisions within the screening program currently have an expert endoscopist who performs en bloc resections and/or surgeons who perform TEM or TAMIS, or appointments with referral centers.

Colonoscopies performed within the screening program are all performed by accredited endoscopists. However, no data were available on whether colonoscopies for local excision or colonoscopies outside of the screening program were performed by these accredited endoscopists or by general endoscopists. Furthermore no data were available on the type of center where local excisions were performed. This might introduce some bias in the results, as accredited screening endoscopists are also likely to perform colonoscopies outside of the screening setting, which implies equal expertise in the local treatment of screen-detected and non-screen-detected CRCs. Quality control measures set in the screening program might therefore also be imposed for endoscopies performed outside of a screening setting. Quality control measures should at least include whether an en bloc resection was performed and details about radicality (R0 /R1 resection).

Long-term recurrence rates of locally excised T1Nx CRCs should confirm whether the decision for local excision only was justified, although a previous population-based study by Senore et al. suggested no difference in recurrence-free survival between local excision only vs. surgical oncologic resection for pT1 tumors with low risk features 11 .

Another implication of the study is that the assessment of stage migration through population-based screening should not rely solely on TNM staging, as a large difference in treatment choice was observed between T1 and T2 stage CRCs. Subgrouping based on T and N classification may provide additional information that can facilitate in-depth evaluation of treatment patterns and outcomes in terms of CRC incidence and CRC-related mortality.

In conclusion, our findings support the idea that the higher level of less invasive treatment for screen-detected stage I CRCs can be attributed, at least in part, to the higher rate of T1 tumors in screen-detected stage I CRCs compared with non-screen-detected cases after adjusting for location, LVI presence, and tumor differentiation. Nevertheless, there are other factors that may account for the discrepancy in treatment between screen-detected and non-screen-detected cases that remain unclear. Future research should investigate if the choice of local excision was related to unidentified cancer-related factors or the expertise of the endoscopists. In the long term, recurrence rates should confirm whether the choice of less invasive treatment was justified.

## Notes

Correction **Correction: Differences in treatment of stage I colorectal cancers: a population-based study of colorectal cancers detected within and outside of a screening program** Toes-Zoutendijk E, Breekveldt ECH, van der Scheeet L al. Differences in treatment of stage I colorectal cancers: a population-based study of colorectal cancers detected within and outside of a screening program. Endoscopy, DOI 10.1055/a-2173-5989. In the above-mentioned article, the name of the author Esther Toes-Zoutendijk has been corrected. This was corrected in the online version on November 28, 2023.

## Table 1 s

Supplementary material Zusatzmaterial
