
JOURNAL INFORMATION
==============================
NLM Title Abbreviation: Endoscopy
Journal ID: Endoscopy
NLM Journal ID: 0215166

Endoscopy

ISSN: 0013-726X
EISSN: 1438-8812

ARTICLE INFORMATION
==============================
PMCID: PMC12419960
PMID: 38016633
DOI: 10.1055/a-2217-6054
Article version: 1
Subjects: Correction

Correction: Differences in treatment of stage I colorectal cancers: a population-based study of colorectal cancers detected within and outside of a screening program

http://orcid.org/0000-0003-4001-2949 Toes-Zoutendijk Esther 1 ‡
Breekveldt Emilie C. H. 1 2 ‡
van der Schee Lisa 3
Nagtegaal Iris D. 4
Elferink Marloes A. G. 5
Lansdorp-Vogelaar Iris 1
Moons Leon M. G. 3
van Leerdam Monique E. 2 6
1 Department of Public Health, Erasmus University Medical Center, Rotterdam, The Netherlands
2 Department of Gastrointestinal Oncology, Netherlands Cancer Institute – Antoni van Leeuwenhoek Hospital, Amsterdam, The Netherlands
3 Department of Gastroenterology and Hepatology, University Medical Center Utrecht, Utrecht, The Netherlands
4 Department of Pathology, Radboud University Medical Center, Nijmegen, The Netherlands
5 Department of Research and Development, Netherlands Comprehensive Cancer Organization, Utrecht, The Netherlands
6 Department of Gastroenterology and Hepatology, Leiden University Medical Center, Leiden, The Netherlands
Corresponding author Esther Toes-Zoutendijk, MD, PhD Department of Public Health, Erasmus University Medical CenterBuilding NA-24 room 18Dr. Molewaterplein 403015 GD, RotterdamThe Netherlandse.toes-zoutendijk@erasmusmc.nl
Electronic publication date: 2023 Nov 28
Publication date: 2024 Jan
Volume: 56
Issue: 1
First page: C11
Last page: C11

Copyright: The Author(s). This is an open access article published by Thieme under the terms of the Creative Commons Attribution License, permitting unrestricted use, distribution, and reproduction so long as the original work is properly cited. (https://creativecommons.org/licenses/by/4.0/)
Copyright year: 2023
Copyright holder: The Author(s).
License: This is an open-access article distributed under the terms of the Creative Commons Attribution License, which permits unrestricted use, distribution, and reproduction in any medium, provided the original work is properly cited.
License URL: https://creativecommons.org/licenses/by/4.0/

Erratum for: Differences in treatment of stage I colorectal cancers: a population-based study of colorectal cancers detected within and outside of a screening program 56 1 7 11 2023 5 13 Endoscopy 10.1055/a-2173-5989 PMC10736105 37935373

==============================
Correction

Correction: Differences in treatment of stage I colorectal cancers: a population-based study of colorectal cancers detected within and outside of a screening program Toes-Zoutendijk E, Breekveldt ECH, van der Scheeet L al. Differences in treatment of stage I colorectal cancers: a population-based study of colorectal cancers detected within and outside of a screening program. Endoscopy, DOI 10.1055/a-2173-5989 . In the above-mentioned article, the name of the author Esther Toes-Zoutendijk has been corrected. This was corrected in the online version on November 28, 2023.

‡ Joint first authors

