# Supplementary material for: Differences in treatment of stage I colorectal cancers: a population-based study of colorectal cancers detected within and outside of a screening program
Source: Endoscopy. 2023 Nov 7;56(1):5–13. doi: 10.1055/a-2173-5989 (PMC10736105; doi:10.1055/a-2173-5989)
Supplement: Supplementary file 1 — Supplementary material [file 22873supmat_10-1055-a-2173-5989.pdf]

Supplementary material

Differences in treatment of stage I colorectal cancers: a population-based study of colorectal cancers detected within and outside of a screening program

Esther Toes-Zoutenkijk, Emilie C. H. Breekveldt, Lisa van der Schee, Iris D. Nagtegaal, Marloes A. G. Elferink, Iris Lansdorp-Vogelaar, Leon M. G. Moons, Monique E. van Leerdam

Table 1s *T-stage distribution and treatment of all T1 CRCs diagnosed in the screening era*

	Screen-detected T1 CRC	Non-screen-detected T1 CRC	*p* value
**T-stage distribution**			0.81
All T1 CRCs, n	4,445	5,800	
T1 stage I CRCs, n (%)	4,167 (93.7)	5,445 (93.9)	
T1 stage III CRCs, n (%)	278 (6.3)	355 (6.1)	
**Treatment all T1 CRCs, n**			<0.0001
Local excision, n (%)	2,543 (57.2)	2,527 (43.6)	
Surgical oncologic resection, n(%)	1,902 (42.8)	3,273 (56.4)	
**Treatment T1 stage I CRCs**			<0.0001
Local excision, n (%)	2,537 (60.9)	2,514 (46.2)	
Surgical oncologic resection, n(%)	1,630 (39.1)	2,931 (53.8)	
**Treatment T1 stage III CRCs**			0.39
Local excision, n (%)	6 (2.2)	13 (3.7)	
Surgical oncologic resection, n(%)	272 (97.8)	342 (96.3)	
